# Phenotypic and Genomic Characterization of Novel Straboviridae Bacteriophages Targeting Multidrug-Resistant *Salmonella enterica* subspecies *enterica* Serovar Enteritidis

**DOI:** 10.3390/microorganisms14061213

**Published:** 2026-05-27

**Authors:** Elias D. Antoun, Salman A. Almashtoub, Gabriel H. Fares, Tasnime A. Abdo Ahmad, Ghassan M. Matar, Esber S. Saba

**Affiliations:** 1 Department of Biomedical Engineering, Maroun Semaan Faculty of Engineering and Architecture, American University of Beirut, Beirut 1107 2020, Lebanon; eda03@mail.aub.edu; 2 Department of Experimental Pathology, Immunology & Microbiology, Faculty of Medicine, American University of Beirut, Beirut 1107 2020, Lebanon; sa109@aub.edu.lb (S.A.A.); gf08@aub.edu.lb (G.H.F.); taa53@mail.aub.edu (T.A.A.A.); 3 Department of Biology, Faculty of Sciences I, Lebanese University, Beirut P.O. Box 6573, Lebanon; 4 Center of Infectious Diseases Research, Faculty of Medicine, American University of Beirut, Beirut 1107 2020, Lebanon; 5 WHO CC for Reference & Research on Bacterial Pathogens, Faculty of Medicine, American University of Beirut, Beirut 1107 2020, Lebanon

**Keywords:** bacteriophage, therapy, *Salmonella enterica*, multidrug resistance, biocontrol

## Abstract

*Salmonella enterica* serovar Enteritidis is a leading cause of foodborne zoonoses worldwide. The rapid emergence of multidrug-resistant (MDR) strains has compromised traditional antimicrobial therapies, necessitating the development of biosafe alternatives such as bacteriophages. This study aimed to isolate and comprehensively characterize novel lytic bacteriophages targeting multidrug-resistant *Salmonella enterica* subspecies *enterica* serovar Enteritidis isolates from Lebanon. In this study, four novel *Salmonella* phages, EDA02, EDA03, EDA05, and EDA06, were isolated from wastewater and poultry effluents in Lebanon. The isolates were characterized using host range profiling, one-step growth kinetics, and physicochemical stability assays. Comprehensive whole-genome sequencing (WGS) and phylogenetic analyses were performed to assess their genomic safety and taxonomic placement. Phages EDA03 and EDA06 exhibited the broadest intra-serovar lytic activity within the tested panel, infecting up to 72% and 67% of the MDR isolates, respectively. One-step growth analysis revealed latent periods of 30–40 min, with burst sizes ranging from 6.0 to 150 phages/infected cell. All four phages demonstrated robust stability across pH 4.7–10.3 and temperatures from 4 °C to 50 °C. WGS revealed genome sizes ranging from 42.3 kb to 108.8 kb, with no identified genes associated with lysogeny, virulence, or antimicrobial resistance. Phylogenomic analysis assigned all isolates to the family Straboviridae, with <95% intergenomic similarity to their closest RefSeq relatives, supporting their classification as novel species. The isolated phages demonstrate substantial lytic activity and environmental resilience under the tested conditions. Their complementary lytic profiles, environmental resilience, and genomic safety support their further evaluation as biocontrol candidates. This study represents the first genomic and phenotypic characterization of anti-*Salmonella* Enteritidis phages from Lebanon. These findings support the development of phage-based interventions for food safety and antimicrobial resistance mitigation in resource-limited settings.

## 1. Introduction

*Salmonella enterica* remains a critical global threat to food safety and public health [1]. Despite intensive monitoring programs, salmonellosis is consistently ranked as one of the most prevalent foodborne zoonoses, accounting for approximately 95 million cases of gastroenteritis annually [2]. This imposes a substantial socioeconomic burden on healthcare systems and the global food industry [3]. Human infection primarily occurs through the consumption of contaminated poultry products, such as meat and eggs, which serve as the main vectors for zoonotic transmission [4]. In poultry systems, *Salmonella* persists through both horizontal and vertical transmission pathways [5]. Historically, the control of *Salmonella* in poultry operations has relied on biosecurity, vaccination, and the prophylactic use of antibiotics [6]. However, decades of excessive antimicrobial use in both veterinary and human medicine have accelerated the emergence and dissemination of antimicrobial resistance (AMR) [7]. Currently, *S.* Enteritidis and *S.* Typhimurium are recognized as major multidrug-resistant (MDR) lineages with significant zoonotic potential [8]. Among these, *S.* Enteritidis, particularly sequence type 11 (ST11), represents the dominant lineage associated with human infections and foodborne outbreaks in the Middle East, including Lebanon [9,10]. Accordingly, the present study focuses specifically on MDR *S*. Enteritidis ST11 isolates as a clinically and regionally relevant model. The World Health Organization (WHO) classifies AMR as a top-tier public health crisis, with forecasts suggesting that drug-resistant infections could cause over 10 million annual deaths by 2050 if novel interventions are not implemented [11]. In the agricultural sector, the non-therapeutic use of antibiotics for growth promotion further exacerbates the crisis, as resistance genes are horizontally transferred through the food chain and into the environment [12]. Current strategies for controlling *S*. Enteritidis in poultry and food-production systems include biosecurity measures, sanitation programs, vaccination, surveillance, and antimicrobial treatment when clinically indicated [13]. However, these approaches have important limitations. Biosecurity and sanitation reduce contamination risk but may not fully eliminate persistent *Salmonella* reservoirs, while antibiotic-based control is increasingly compromised by the emergence of multidrug-resistant strains [14,15]. These limitations highlight the need for alternative, targeted, and environmentally sustainable approaches, among which bacteriophage-based biocontrol has gained increasing attention.

Given this global threat, the development of sustainable, antibiotic-free interventions is imperative. Bacteriophages, viruses that specifically infect and lyse bacterial hosts without disrupting the native microbiota, represent a highly promising alternative [16]. Phages offer several distinct advantages, including high host specificity, self-replication at the site of infection, and a favorable ecological safety profile [11]. Several phage-based biocontrol agents have already attained “Generally Recognized as Safe” (GRAS) status from the U.S. Food and Drug Administration (FDA) for applications in food processing [17]. Nevertheless, the widespread implementation of phage therapy [18] is still hindered by research gaps regarding environmental stability and the long-term sustainability of phage cocktails in preventing the emergence of bacterial resistance [19]. Advancing phage-based biocontrol requires a precise understanding of phage–host interactions, including adsorption kinetics, replication dynamics, and the synergy of complementary lytic activities in cocktail formulations [20]. Furthermore, parameters such as thermal resilience and host range are critical for industrial application, where processing temperatures and contamination sources vary significantly [21].

Therefore, the present study aimed to isolate and comprehensively characterize four novel bacteriophages, EDA02, EDA03, EDA05, and EDA06, targeting MDR *S*. Enteritidis ST11 isolates from Lebanon. The study specifically addressed three major questions: whether the isolated phages exhibit lytic activity against regional MDR isolates, whether they possess physicochemical stability compatible with future biocontrol applications, and whether their genomes lack lysogeny-, virulence-, or antimicrobial-resistance-associated genes. By integrating phenotypic characterization with deep genomic profiling, this research seeks to design effective, thermally stable, and biosafe phage preparations. These findings aim to provide a robust framework for phage-based alternatives to traditional antibiotics, contributing to the global effort to mitigate AMR and ensure the sustainability of human and animal health.

In this context, the present study also addresses an important regional knowledge gap, as limited studies from the Middle East have combined phenotypic characterization with whole-genome safety profiling of phages targeting multidrug-resistant *S. enterica* serovar Enteritidis. Accordingly, we integrated host range analysis, one-step growth kinetics, physicochemical stability testing, and comparative genomics to characterize four newly isolated phages from Lebanon and evaluate their potential as biocontrol candidates.

## 2. Materials and Methods

### 2.1. Bacterial Strains and Growth Conditions

Eighteen multidrug-resistant (MDR) clinical isolates, including one extensively drug-resistant (XDR) isolate of *Salmonella enterica* serovar Enteritidis were used as host strains in this investigation. All isolates belonged to sequence type 11 (ST11), as confirmed by genomic data (Appendix Table A1), and were obtained from a single national surveillance program. The isolates were provided by the Department of Experimental Pathology, Immunology, and Microbiology at the American University of Beirut (AUB), Lebanon, and were originally recovered from a range of clinical specimens.

Primary cultivation of the isolates was carried out on *Salmonella*–Shigella (SS) agar (Oxoid, Basingstoke, UK), followed by subculture on Luria–Bertani (LB) agar or in LB broth (Neogen Miller, Lansing, MI, USA) for routine propagation. All bacterial cultures were incubated at 37 °C with continuous agitation at 160 rpm. For preservation, glycerol stocks (20%, *v*/*v*; Fisher Chemical, Waltham, MA, USA) were prepared and stored at −80 °C. Before each experimental assay, bacterial suspensions were standardized to 0.5 McFarland turbidity, corresponding approximately to 1 × 10^8^ Colony Formation Unit (CFU/mL) [22]. Antimicrobial susceptibility testing was performed against a panel of clinically relevant antibiotics, including ceftriaxone, meropenem, ampicillin, and amoxicillin–clavulanic acid (β-lactams); gentamicin (aminoglycoside); ciprofloxacin and nalidixic acid (fluoroquinolones); tetracycline (tetracyclines); chloramphenicol (phenicols); colistin (polymyxins); trimethoprim–sulfamethoxazole (folate pathway inhibitors); and azithromycin (macrolides). Minimum inhibitory concentration (MIC) values were interpreted according to EUCAST 2026 clinical breakpoints. Multidrug resistance (MDR) was defined as acquired non-susceptibility to at least one agent in three or more antimicrobial classes, while extensively drug-resistant (XDR) isolates were defined as non-susceptibility to all but one or two antimicrobial classes, in accordance with established international criteria. Based on these criteria, only one isolate met the definition of XDR, while all remaining isolates were classified as MDR (Appendix Table A1).

### 2.2. Sewage Sample Collection and Processing

Untreated sewage samples were collected from geographically distinct sites across Lebanon, including municipal wastewater discharge points and poultry farm effluents from Beirut, Mount Lebanon, Bekaa, and South Lebanon. Sampling sites were selected based on their high likelihood of containing enteric bacterial contamination and bacteriophage diversity associated with human and poultry waste. Samples were collected aseptically in sterile Falcon tubes and transported to the laboratory on ice for immediate processing within the same day. Although larger sample volumes are commonly used in environmental virome studies, the present study employed an enrichment-based isolation strategy, allowing successful phage recovery from smaller sample volumes. The chemical composition and physicochemical parameters of the wastewater samples were not analyzed, as the primary objective of this study was bacteriophage isolation and characterization rather than environmental wastewater profiling. Samples were centrifuged at 6400× *g* for 15 min at 4 °C after 18 h of sedimentation at room temperature. Syringe filters with 0.22 µm diameter (Millipore, Darmstadt, Germany) were used to filter the clarified supernatants to remove bacteria remnants. Eight combinations were prepared from the filtered samples to be pre-screened against eight *S.* Enteritidis strains [23,24].

### 2.3. Isolation and Enrichment of Bacteriophages

To conduct the initial screening, 100 µL of each sewage sample combination was mixed with 100 µL of a 0.5 McFarland *Salmonella enterica* suspension and 50 µL of LB broth in sterile 96-well round-bottom plates. Positive control (PC) wells contained 200 µL of host bacterial suspension without sewage filtrate, whereas negative control (NC) wells contained 200 µL of sterile LB broth only. Optical density (OD) measurements were recorded every 30 min at 37 °C using a multimode microplate reader [25]. Wells exhibiting reduced absorbance relative to the positive controls were considered indicative of potential bacteriophage activity. This preliminary enrichment strategy was designed to selectively amplify bacteriophages capable of infecting multidrug-resistant (MDR) *S.* Enteritidis host strains present in the tested sewage samples. Reduced optical density relative to untreated bacterial controls was interpreted as indirect evidence of bacteriolytic activity.

For phage enrichment, 10 mL of the corresponding sewage mixture was combined with 2 mL of a 0.5 McFarland *Salmonella* culture and 2 mL of LB broth, followed by incubation for 18 h at 37 °C with agitation at 150 rpm using an orbital shaking incubator (New Brunswick Scientific, Edison, NJ, USA). The enrichment culture was subsequently centrifuged at 6400× *g* for 15 min at 4 °C, and the supernatant was filtered through 0.22 µm syringe filters to remove residual bacterial cells and debris.

Phage isolation was performed using the double-layer agar (DLA) method. Briefly, 100 µL of filtered lysate was mixed with 1 mL of a 0.5 McFarland host bacterial suspension and 3 mL of molten LB soft agar (0.6% agar), then overlaid onto solid LB agar base plates (1.5% agar). Plates were incubated overnight at 37 °C and examined for plaque formation. Clear plaques were interpreted as evidence of productive lytic infection.

Individual plaques were carefully picked and resuspended in 0.5 mL of SM buffer (50 mM Tris-HCl, pH 7.5, 100 mM NaCl, 8 mM MgSO_4_), followed by centrifugation at 8000× *g* for 5 min. The recovered suspension was subsequently re-enriched with the corresponding host culture in LB broth. This plaque purification process was repeated three consecutive times to ensure clonal purity and eliminate the possibility of mixed-phage populations within the lysates.

Phage titers were determined using the DLA assay and expressed as plaque-forming units per milliliter (PFU/mL). Purified phage lysates were stored at 4 °C until further characterization assays were performed [26]. Countable plates containing 30–300 plaques were used for titer determination according to the following equation [8]:

$$PFU/mL=\frac{number\;of\;plaques\;\times \;Dilution\;factor}{Volume\;plated\;(mL)}$$

### 2.4. Phage Purification and Propagation

The culture in 10 mL of LB broth containing 10 mM of CaCl_2_ to maximize adsorption was further subculture to phage stocks by inoculating exponentially growing *Salmonella* cultures (0.5 McFarland). Calcium chloride supplementation was used to enhance phage adsorption efficiency by facilitating phage attachment to the bacterial surface [27]. A culture incubated at 18 h at 37 °C and 150 rpm was centrifuged (4000× *g*, 15 min) and then filtered (0.22 μm). This process was repeated three times to obtain high-titer lysates. Sequential amplification cycles were performed to increase phage titer and improve lysate purity before downstream characterization assays [28]. The multiplicity of infection (MOI) was calculated using the PFU counts of DLA, according to the following formula [29]:

$$MOI=\frac{number\;of\;infectious\;viral\;particles}{number\;of\;targeted\;bacterial\;cells}$$

MOI was calculated based on experimentally determined PFU/mL and CFU/mL values at the time of infection. CFU/mL values were determined prior to infection to ensure accurate MOI calculation.

### 2.5. Host Range Determination

The host range of the individual bacteriophages was identified using the DLA technique. Ten-fold serial dilutions of the phage lysates were prepared in LB broth. Then, 100 µL of each dilution was mixed with 1 mL of a 0.5 McFarland bacterial suspension (approximately 1 × 10^8^ CFU/mL, standardized using a densitometer) and 900 µL of LB broth. This mixture was combined with 3 mL of molten LB soft agar (0.6%) and overlaid onto 1.5% LB agar base plates. Plates were incubated overnight at 37 °C and examined for plaque formation or lytic zones, which indicated phage activity. The degree of lysis was assessed based on plaque clarity and morphology [30]. Efficiency of plating (EOP) was calculated by comparing PFU on each test strain to PFU on the original host strain [11]. All EOP values were experimentally determined using the DLA method. Briefly, phage lysates were serially diluted and plated on each test strain and on the original propagation host under identical conditions. PFU/mL were calculated for each condition, and EOP values were computed as the ratio of PFU obtained on the target strain to PFU obtained on the original host. All assays were performed in triplicate, and values are presented as mean estimates. All bacterial isolates used in host range determination were previously characterized at the genomic level and assigned to sequence type ST11, as detailed in Appendix Table A1.

### 2.6. One-Step Growth Curve

One-step growth experiments were performed at MOI 0.1. 15 mL of 0.5 McFarland bacterial suspension were prepared from which 900 µL were added to fifteen 1.5 mL microcentrifuge tubes, which were labeled according to the sampling time points (0, 5, 10, 20, 30, 40, 50, 60 and 70 min) while 900 µL LB Broth only was added to the tube labeled 0 min. To synchronize infection, the phage–host mixture was incubated for 10 min at 37 °C to allow for adsorption. Following the 10 min adsorption period, the mixture was centrifuged at 8000× *g* for 5 min. The supernatant containing unadsorbed phages was discarded, and the pellet was washed once with pre-warmed LB broth to minimize carry-over of free virions before final resuspension in 15 mL of LB broth. This washing step ensured that the subsequent rise in PFU/mL accurately represented a single, synchronized burst event. This approach was intended to promote synchronized infection and minimize coinfection events, thereby improving estimation of latent period and burst size.

A low MOI (0.1) was employed to ensure predominantly single infections per bacterial cell, allowing for the accurate calculation of latent period and burst size without the interference of coinfection. Following adsorption and removal of unadsorbed phages, samples were collected at predetermined time intervals (0–70 min), serially diluted, and plated using the DLA method to determine phage titers. All tubes were centrifuged at 8000× *g* for 5 min, supernatants were carefully recovered, placed on ice, and serially diluted and plated into LB agar plates after adding 1 mL 0.5 McFarland bacterial suspension and 3 mL LB top agar (semi-solid 0.6% agar), and incubated overnight at 37 °C. The PFUs were counted to determine PFU/mL at each time point [31].

The burst size, defined as the number of progeny phages produced per infected bacterial cell, was calculated using the following formula:

$$Burst\;size=\;\frac{\frac{PFU}{mL}\;at\;plateau\;phase-\frac{PFU}{mL}at\;end\;of\;the\;latent\;period}{Initial\;number\;of\;infected\;bacterial\;cells}$$

### 2.7. Adsorption Rate Assay

Adsorption assays were performed at MOI 0.1 under the same infection conditions used for the one-step growth experiment, except that samples were collected over 30 min to estimate the proportion of unadsorbed phages remaining in the supernatant. Adsorption efficiency was determined by quantifying the decrease in extracellular, non-adsorbed phage particles remaining in the supernatant over time. Another time control was included, where no bacteria were added; it contained 900 µL of LB Broth and 100 µL of bacteriophage lysate. The tubes were centrifuged at 8000× *g* for 5 min at 4 °C to obtain the supernatant, which was then diluted, plated, and counted [32]. Due to the relatively broad sampling intervals, adsorption kinetics should be interpreted qualitatively rather than as precise kinetic measurements. The percentage of adsorbed phages was calculated relative to the no-bacteria control at each time point.

### 2.8. Bacteriolytic Activity

To evaluate bacteriolytic performance, four MOIs (10, 1, 0.1, 0.01) were tested for each phage. In 96-well plates, 75 µL of 0.5 McFarland *Salmonella* suspension was mixed with 105 µL of phage lysate. Multiple MOIs were tested to evaluate the influence of infection pressure on bacterial suppression dynamics and phage replication efficiency [33]. OD_600_ readings were recorded every 30 min for 12 h at 37 °C using a multimode plate reader. LB broth served as the negative control, while untreated bacterial cultures served as positive control [34]. Optical density measurements were used as an indirect indicator of bacterial growth kinetics and were interpreted alongside CFU and PFU quantification to improve biological interpretation [35]. In parallel, bacterial viability was quantified by plating serial dilutions at selected time points to determine CFU/mL, and phage replication was assessed by plaque assays to determine PFU/mL. Samples were collected at defined intervals, serially diluted in LB broth, and plated using standard microbiological techniques. All measurements were performed in triplicate.

### 2.9. pH and Thermal Stability

pH stability was determined using buffered LB broth adjusted to different pH values. 100 µL of phage lysate were added to 900 µL of buffered LB Broth and incubated for 2 h at 37 °C, followed by a conventional plaque assay to determine the PFU count. The thermal stability of the purified phage lysates was also tested to determine their sensitivity to variations in temperature that are pertinent in food processing and in the environment. Phage suspensions were then diluted to an MOI of 10 and were diluted in sterile LB broth and maintained at neutral pH. Control thermal treatments were applied to the aliquots of 250 µL that were transferred to sterile microcentrifuge tubes using a calibrated dry-block thermostat [8]. Acidic pH conditions (pH 2–6) were prepared using phosphate–citrate buffer systems, whereas neutral to alkaline conditions (pH 7–13) were adjusted using Tris-based buffer systems to ensure stable pH conditions throughout the incubation period.

Refrigeration, ambient, physiological, and moderate heat-stress conditions were simulated by the incubation of samples at 4 °C, 25 °C, 37 °C, 50 °C, 60 °C, and 70 °C for 2 h. A fixed incubation period of 2 h was selected to evaluate endpoint physicochemical stability under standardized exposure conditions rather than time-dependent inactivation kinetics [36]. All the samples were cooled down to ice after incubation to prevent further thermal inactivation [11]. Following exposure, all samples were immediately cooled on ice prior to plaque assay quantification in order to minimize continued thermal inactivation during handling.

The remaining phage activity was determined by the number of colony count on the DLA assay against *Salmonella enterica* serovar Enteritidis (0.5 McFarland). PFU/mL was counted three times at each temperature condition. Relative stability was determined as a percentage of the initial titer measured before treatment [37]. Stability was assessed after a fixed exposure period of 2 h under each condition; therefore, the results represent endpoint measurements rather than time-resolved kinetic inactivation profiles.

### 2.10. Phage DNA Extraction

Phage genomic DNA was extracted using a modified phenol–chloroform–isoamyl alcohol method. Briefly, phage lysates were incubated with DNase I (5 µL, 20 mg/mL; Roche Diagnostics, 11284932001, Basel, Switzerland) and RNase A (1 µL, 10 mg/mL; Roche Diagnostics, 11119915001, Basel, Switzerland) to eliminate contaminating host nucleic acids, followed by enzyme inactivation using 20 µL of 0.5 M EDTA (Merck, E7889, Darmstadt, Germany). Capsid proteins were subsequently digested using Proteinase K (2 µL, 20 mg/mL; Hilden, Germany) in the presence of SDS (50 µL; Merck, 428018, Darmstadt, Germany). An equal volume of phenol:chloroform:isoamyl alcohol (25:24:1) (Merck, 136112-00-0, Darmstadt, Germany) was then added, and the mixture was centrifuged to separate the aqueous phase. The recovered supernatant was further purified using chloroform:isoamyl alcohol (24:1) (Merck, C0549, Darmstadt, Germany), followed by an additional centrifugation step. DNA precipitation was performed using 3 M sodium acetate (1:10 volume; Merck, 127-09-3, Darmstadt, Germany) and 2.5 volumes of absolute ethanol (Merck, 64-17-5, Darmstadt, Germany). The resulting DNA pellet was washed with 200 µL of 70% ethanol and resuspended for downstream analysis. DNA concentration and purity were evaluated using a Qubit 4.0 Fluorometer (Thermo Fisher Scientific, Waltham, MA, USA) and a NanoDrop spectrophotometer to ensure adequate high-molecular-weight DNA quality for long-read sequencing applications. To further confirm the absence of lysogenic signatures, predicted proteins were screened for conserved integrase- and excisionase-associated domains using the Pfam and PHROGs databases [11].

### 2.11. Comparative Genomics, Phylogenetic and Bioinformatics Analysis

Phage genomic DNA libraries were prepared according to the manufacturer’s protocol (Oxford Nanopore Technologies, Oxford, UK) and sequenced using long-read sequencing on a MinION platform (Oxford Nanopore Technologies) at the DNA Sequencing Facility, American University of Beirut.

Assembly and annotation of the genomes of phages EDA02, EDA03, EDA05, and EDA06 were performed in a Linux environment using command-line tools. Raw reads were quality-filtered and assembled using Flye (v2.9.3), which produced phage genomes solved as single contigs with an average depth resolution of more than 50×. The phage genome was annotated using Pharokka (https://usegalaxy.eu/root?tool_id=pharokka, accessed on 26 November 2025), a command-line pipeline for phage genome annotation. Pharokka combines various bioinformatics pipelines, including BLASTx 2.15.0 searches against phage-specific databases (PHROGs, UniProt, Pfam), conserved domain prediction, and protein structural prediction. The genomes of the phages were further analyzed using PhageScope (https://phagescope.deepomics.org, accessed on 26 November 2025) [38], a bioinformatic tool used to predict phage lifestyle-associated features and screen for genes linked to lysogeny, virulence, and AMR. To improve annotation robustness, predicted phage proteins were further cross-validated by manual inspection against PHROGs, UniProt, and Pfam conserved-domain databases. Safety screening focused specifically on identifying canonical lysogeny markers, virulence-associated genes, and phage-borne antimicrobial resistance determinants.

Taxonomy of the phage was established with BLASTn (https://blast.ncbi.nlm.nih.gov/Blast.cgi, accessed on 26 November 2025) against the NCBI database, and phylogenomic distances were rated with the help of VIRIDIC (Virus Intergenomic Distance Calculator, https://bio.tools/viridic, accessed on 27 November 2025) using the 95% intergenomic similarity threshold for species-level demarcation [39]. ViPTree (v4.0) was used to construct a phylogenetic tree of similarities in the genome sequences [40]. ViPTree makes use of the Genome-BLAST Distance Phylogeny (GBDP) (https://ggdc.dsmz.de, accessed on 27 November 2025) approach to compute intergenomic distances. The GBDP approach compares genomes with the help of BLAST, and the distances are calculated depending on the presence and length of identical fragments. In particular, ViPTree provides the BLASTn application on the nucleotide level.

A phylogenetic tree was constructed on the distance matrix that was produced by the GBDP, and the tree was constructed using the Fast ME algorithm, which constructs trees using the balanced minimum evolution criterion, which ensures the creation of a stable and strong phylogenetic relationship. EDA02, EDA05, EDA03, EDA06 and their closely related phages also formed a comparative map constructed using ViPTree. This comparative analysis highlights the major similarities and differences in genomic features among the identified bacteriophages and was facilitated using PhageScope, a dedicated platform for bacteriophage genome comparison and visualization [38].

To confirm the absence of lysogenic potential, predicted proteins were screened for canonical lysogeny markers, including integrases, excisionases, recombinases, and CI-like repressors, using PHROGs, UniProt, and Pfam databases. Anti-repressor-like annotations, when present, were interpreted conservatively and were not considered sufficient evidence of temperate potential in the absence of core lysogeny machinery. No canonical lysogeny-associated genes were identified in any of the four phages [41].

### 2.12. Data Presentation and Statistical Considerations

All experiments were performed in three independent biological replicates unless otherwise stated, and results are presented as mean ± standard deviation (SD). The present study was primarily designed as a phenotypic and genomic characterization study. Data organization and visualization were performed using Python (v3.12). Phage stability under different pH and temperature conditions was analyzed using two-way ANOVA, with phage identity and treatment condition as fixed factors. When a significant interaction or main effect was detected, post hoc multiple comparisons were performed using Dunnett’s test against the reference condition: pH 7 for the pH stability assay and 37 °C for the thermal stability assay. Data are presented as mean ± SD from three independent biological replicates, and *p* < 0.05 was considered statistically significant.

## 3. Results

### 3.1. Isolation of Four Salmonella Phages

**Figure 1:**
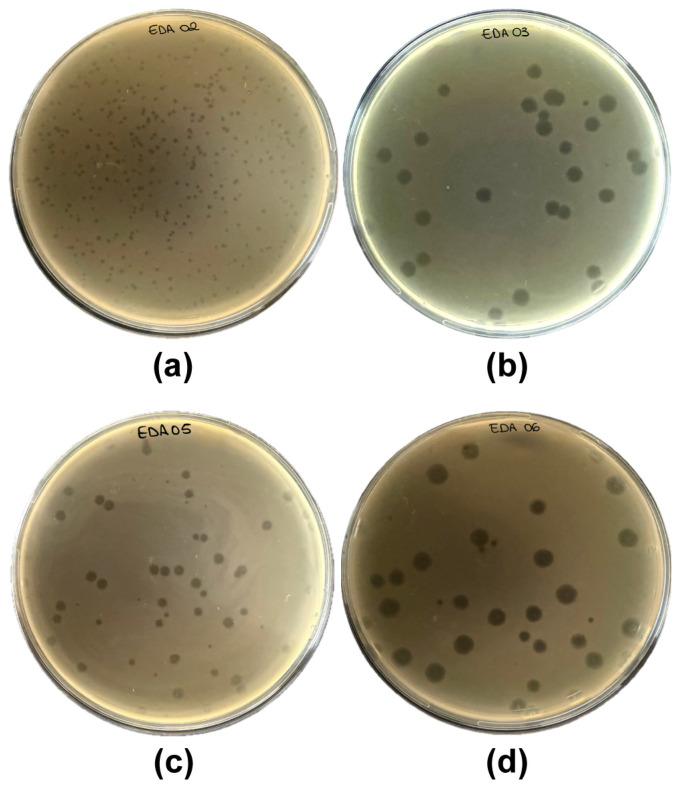
Plaque morphology of *Salmonella* phages EDA02, EDA03, EDA05, and EDA06. Representative agar plates showing plaque formation following infection of the bacterial host with four distinct bacteriophages. (**a**) EDA02 displays numerous small, pinpoint plaques distributed uniformly across the bacterial lawn. (**b**) EDA03 forms fewer but larger, well-defined plaques with clear zones of lysis. (**c**) EDA05 exhibits plaques of heterogeneous sizes, suggesting variability in lytic activity or diffusion. (**d**) EDA06 produces relatively large, round plaques with clear boundaries. No halo zones were observed around the plaques for any of the four isolates. Differences in plaque size and morphology reflect phage-specific lytic properties and infection dynamics. Representative images from three independent experiments.

*Salmonella* phages EDA02, EDA03, EDA05, and EDA06 were isolated from untreated sewage samples collected from geographically diverse sites in Lebanon, including municipal wastewater discharge points and poultry farm effluents. Each phage was isolated using a specific multidrug-resistant (MDR) *Salmonella enterica* serovar Enteritidis clinical host: EDA02 was isolated on strain SAL282, EDA05 on SAL256, EDA03 on SAL240, and EDA06 on SAL242. Clear, circular-shaped plaques were observed for all four isolates on DLA plates (Figure 1). To ensure clonal purity and stability, each phage was enriched and purified on its respective host at least three consecutive times. All four phages formed distinct clear lytic zones on the bacterial lawn, consistent with productive infection. Plaques ranged from pinpoint morphology to larger, well-defined clear plaques.

### 3.2. Phage Host Range Determination

**Figure 2:**
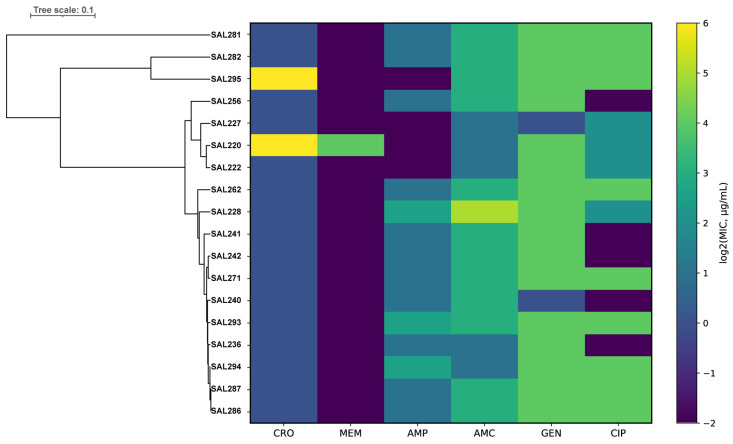
Hierarchical clustering and antimicrobial susceptibility profiles of multidrug-resistant *Salmonella enterica* serovar Enteritidis isolates. The dendrogram (**left**) represents hierarchical clustering of clinical isolates based on similarity in their antimicrobial resistance patterns. The heatmap (**right**) displays the log2-transformed minimum inhibitory concentration (MIC) values for six antibiotics: ceftriaxone (CRO), meropenem (MEM), ampicillin (AMP), amoxicillin–clavulanic acid (AMC), gentamicin (GEN), and ciprofloxacin (CIP). Color intensity reflects resistance levels, with higher values (yellow/green) indicating increased resistance and lower values (purple/blue) indicating susceptibility. The clustering reveals heterogeneity in resistance profiles across isolates, despite belonging to the same serovar, highlighting the multidrug-resistant nature of the tested panel.

We tested the host range of *Salmonella* phages EDA02, EDA03, EDA05, and EDA06 against 18 multidrug-resistant (MDR) *Salmonella* Enteritidis clinical strains. These strains, originally recovered from clinical specimens, all belong to sequence type 11 (ST11), the dominant lineage associated with *S.* Enteritidis infections in the region. While this genetic homogeneity ensures consistency in host–phage interaction analyses, it also limits the ability to extrapolate findings across other sequence types or serovars. Tested strains’ antimicrobial resistance profiles are shown in Figure 2. Phages EDA03 and EDA06 showed the broadest lytic activity within the tested isolate panel, demonstrated by their ability to infect 72% and 67% of the tested MDR isolates, respectively, spanning different clusters within the panel. Specifically, EDA03 lysed 13/18 isolates and EDA06 lysed 12/18 isolates. In contrast, phages EDA02 and EDA05 exhibited narrower host spectra, demonstrating high specificity toward their original isolation hosts and a few related strains. Within the tested collection, these data indicate relatively broad intra-serovar activity for EDA03 and EDA06, although broader host-range claims require validation against a larger and geographically diverse strain panel.

Detailed antimicrobial susceptibility profiles, including minimum inhibitory concentration (MIC) values and categorical interpretations, are provided in Appendix Table A1. The isolates exhibited heterogeneous resistance patterns across multiple antibiotic classes, with frequent resistance observed to fluoroquinolones, aminoglycosides, and β-lactams. Notably, phage susceptibility was retained across isolates with diverse resistance profiles, including those exhibiting multidrug-resistant (MDR) and extensively drug-resistant (XDR) phenotypes.

**Figure 3:**
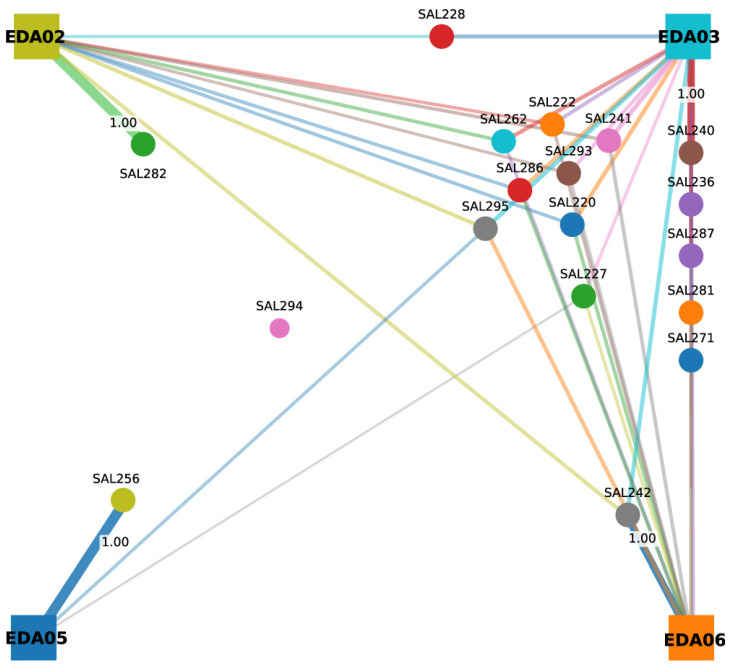
Phage–host proximity network based on EOP values across tested *Salmonella* strains. Square nodes represent bacteriophages, whereas circular nodes represent bacterial strains. Edges connect each phage to strains with measurable EOP values. Edge thickness is proportional to EOP magnitude, with thicker edges indicating higher plating efficiency. Strains without measurable EOP values are shown without phage association.

Additionally, phage virulence was assessed by the EOP, which is the ratio of the average PFU on target bacteria to the average PFU on the original host bacteria. The complete numerical EOP dataset, expressed as mean ± SD from three independent experiments, is provided in Appendix Table A2. Based on EOP values, phage virulence was categorized as follows: EOP ≥ 0.5 (highly virulent), 0.1–0.5 (moderately virulent), 0.001–0.1 (slightly virulent), and ≤0.001 (low virulence) [42] (Figure 3). EOP values were derived from quantitative plaque assays performed under standardized conditions and represent mean values from three independent experiments.

Together, these findings indicate distinct host range profiles among the four phages within the tested ST11 panel. Given the limited size and genetic homogeneity of the isolate set, host range patterns should be interpreted as qualitative trends rather than definitive phylogeny–susceptibility relationships.

### 3.3. Phage One-Step Growth and Adsorption Rate

**Figure 4:**
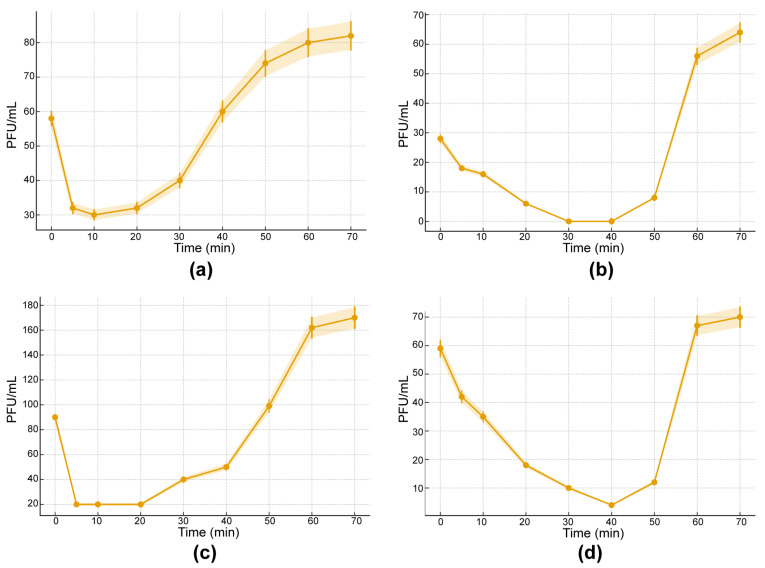
One-step growth curves of bacteriophages infecting *Salmonella* spp. One-step growth analysis showing phage titer changes over time following synchronized infection of the bacterial host. Phage titers (PFU/mL) were determined at the indicated time points following infection of the bacterial host under synchronized conditions. (**a**) EDA02, (**b**) EDA03, (**c**) EDA05, and (**d**) EDA06. The initial decline in extracellular phage counts corresponds to the adsorption phase, followed by a latent period during which no infectious particles are detected in the medium. The subsequent rapid increase in phage titer reflects the burst phase and release of progeny virions. Differences in latent period duration and burst magnitude highlight phage-specific replication dynamics. Data represent mean ± SD of three independent experiments; where error bars are not visible, they fall within the symbol size.

The adsorption rates and one-step growth curves of phages EDA02, EDA03, EDA05, and EDA06 were evaluated using their respective *Salmonella* Enteritidis hosts to characterize their infection dynamics (Figure 4). All four phages exhibited high affinity for their host cells, with approximately 60–80% of the initial phage particles adsorbing within the first 10 min of incubation. This rapid reduction in free phage titers (Figure 4a–d, T = 0 to T = 10 min) indicates efficient recognition of the bacterial surface receptors, followed by a stabilization period before the onset of the latent phase.

The one-step growth curves revealed distinct replication parameters among the isolates. Burst size was calculated from the increase in phage titer between the end of the latent period and the plateau phase relative to the initial number of infected cells. Phages EDA03 and EDA06 (Figure 4b,d) exhibited a relatively longer latent phase of approximately 40 min, followed by a sharp burst phase where the phage titers peaked at approximately 70 min. In contrast, EDA02 and EDA05 (Figure 4a,c) showed a shorter latent period of roughly 30 min, with progeny release initiating earlier and reaching a plateau by 60 min.

Compared to previously reported *Salmonella* phages exhibiting burst sizes between 20 and 50 PFU/cell, EDA05 demonstrated the highest replication efficiency with a burst size of 150 phages/infected cell, while EDA02 yielded 52 phages/infected cell. Phages EDA03 and EDA06 exhibited lower burst sizes but comparable latent periods under the tested conditions, with burst sizes of 6 and 7 phages/infected cell, respectively. EDA02 and EDA05 showed higher progeny production, whereas EDA03 and EDA06 showed lower burst sizes under the tested conditions. Because adsorption was sampled at relatively broad time intervals, the adsorption data should be interpreted as qualitative rather than as high-resolution kinetic measurements. All values are presented as mean ± SD from three independent experiments. Because the experiments were intended for comparative phenotypic characterization, the results are interpreted descriptively rather than through formal inferential statistical testing.

### 3.4. Bacterial Growth Suppression and Phage Replication Dynamics

**Figure 5:**
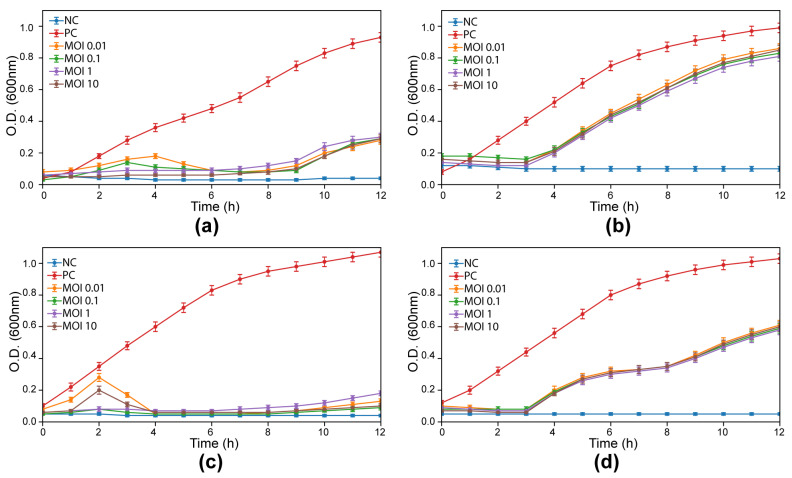
Effect of bacteriophage infection on the growth of MDR *Salmonella* strains at different multiplicities of infection. Bacterial growth was monitored by measuring optical density at 600 nm (OD_600_) over time following infection with bacteriophages at increasing MOIs. (**a**): Phage EDA02, (**b**): Phage EDA03, (**c**): Phage EDA05, (**d**): Phage EDA06. Growth curves of four representative MDR *Salmonella* isolates exposed to the corresponding phages. Non-infected bacteria (positive control, PC) show normal exponential growth, whereas the negative control (NC) remained stable throughout the experiment. Phage treatment reduced bacterial growth relative to untreated controls, although inhibition patterns differed modestly across MOIs and varied primarily according to phage identity. Partial bacterial regrowth was observed after approximately 5 h for EDA03 and EDA06 across multiple MOIs despite sustained phage replication, suggesting the emergence of resistant or tolerant bacterial subpopulations during prolonged incubation. Data represent mean ± SD of three independent experiments; error bars may be smaller than the symbol size.

The bacteriolytic efficacy of *Salmonella* phages EDA02, EDA03, EDA05, and EDA06 was evaluated at multiple MOIs (10, 1, 0.1, and 0.01) against their respective clinical *Salmonella* Enteritidis hosts. As depicted in Figure 5a–d, the dynamics of bacterial growth were monitored by measuring the optical density (OD_600_) over a 12 h incubation period. To validate these observations, bacterial viability and phage replication were quantified in parallel using CFU/mL and PFU/mL measurements. For all four host strains, the positive controls (PC; host without phage) exhibited robust exponential growth, characterized by high carrying capacities (average k = 1.150) and steady growth rates (average r = 0.785). In contrast, no significant growth was detected in the negative controls (NC; sterile broth).

Upon phage treatment, bacterial growth was suppressed relative to untreated controls, although the magnitude and duration of inhibition varied among phages and MOIs. For phages EDA03 and EDA06, bacterial growth was strongly suppressed across all tested MOIs (10 to 0.01), particularly during the early and mid-incubation period, before partial regrowth became evident. However, bacterial recovery was observed after approximately 5 h, suggesting the emergence of phage-resistant subpopulations. Similarly, phages EDA02 and EDA05 reduced bacterial growth across all tested MOIs, with somewhat greater early suppression observed at MOIs 10 and 1 relative to lower MOIs. While a minor increase in OD_600_ was noted at the lowest MOIs (0.1 and 0.01) toward the end of the 12 h period, potentially indicating the emergence of phage-resistant subpopulations, the carrying capacities remained substantially lower than the positive controls. However, complete dose-dependent inhibition was not observed, as inhibition profiles across different MOIs were broadly comparable and bacterial regrowth occurred at later time points under several conditions. This late regrowth may reflect the emergence of phage-resistant bacterial subpopulations or incomplete lysis at low MOIs. CFU/PFU enumeration supported the OD_600_-based observations. At higher MOIs, viable bacterial counts initially stabilized or decreased relative to untreated controls, while PFU/mL increased, indicating active phage replication. At later time points, partial CFU regrowth was observed despite sustained or increasing phage titers, particularly at lower MOIs, suggesting that high extracellular phage abundance did not necessarily correspond to complete bacterial eradication. This phenomenon likely reflects the emergence of phage-resistant or phage-tolerant bacterial subpopulations, together with dynamic phage–host coexistence during prolonged incubation (Appendix Table A3).

### 3.5. Physicochemical Stability of the Isolated Phages

**Figure 6:**
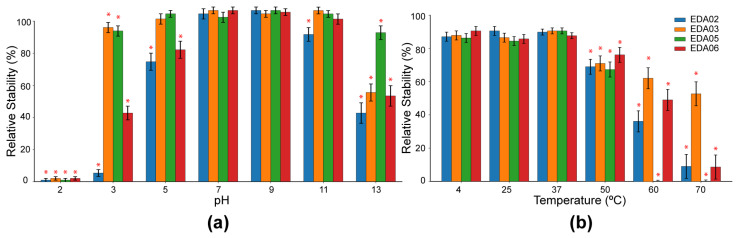
pH and temperature stability of the isolated bacteriophages. The stability of bacteriophages EDA02, EDA03, EDA05, and EDA06 was evaluated under different physicochemical conditions and expressed as relative stability (%). (**a**) pH stability assay showing phage viability after exposure to a range of pH values (pH 2–13). All phages remained highly stable under neutral to mildly alkaline conditions, with reduced stability observed at extreme acidic and alkaline pH. (**b**) Thermal stability assay showing phage viability following incubation at different temperatures (4–70 °C). Asterisks indicate significant differences compared with the reference condition: pH 7 for pH stability and 37 °C for thermal stability, using two-way ANOVA followed by Dunnett’s multiple-comparison test. * *p* < 0.05. The phages maintained high stability at low to moderate temperatures, whereas elevated temperatures resulted in a marked decline in viability, with phage-specific differences in thermal tolerance. Data are presented as bar charts representing discrete test points; error bars indicate the standard deviation of three independent replicates.

The four phages displayed distinct infectivity patterns across the tested pH range (2.34–13), as shown in Figure 6a. At strongly acidic pH values (2.34–3.34), all phages showed minimal or undetectable titers. EDA02 remained relatively stable from pH 4.7 to 10.0, with a progressive decline at pH 11.3–13. EDA03 retained stable infectivity between pH 4.7 and 10.3, followed by reduced titers under more alkaline conditions. EDA05 exhibited a narrower stability range, remaining viable mainly between pH 4.7 and 10.3. EDA06 showed comparatively better tolerance under mildly acidic conditions and remained stable between pH 3.7 and 11.3. Overall, all phages maintained detectable infectivity primarily within the pH range of 4.7–10.3, with EDA06 showing slightly extended tolerance toward mildly acidic conditions (down to pH 3.7) and EDA02 retaining partial stability under more alkaline conditions (up to pH 11.3).

To determine the suitability of the four isolated phages in various environmental and food-processing conditions, the thermal tolerance of the phages was tested over 4–70 °C for 2 h. Figure 6b indicates that all phages were stable with high titers at 4 °C to 50 °C, indicating strong stability under storage and physiologically relevant temperatures.

EDA02 and EDA05 showed almost no change in infectivity to 50 °C, with only a slight decrease in infectivity occurring between 60 °C and 70 °C, after which a sharp decrease in infectivity was observed at 70 °C. Likewise, EDA03 and EDA06 had a similar behavior, maintaining approximately 70–80% of their initial titers at 50 °C but with a significant decline at high temperatures, above 60 °C. Results are shown as mean ± SD of three independent experiments.

### 3.6. Whole-Genome Sequencing and Bioinformatic Analysis

**Figure 7:**
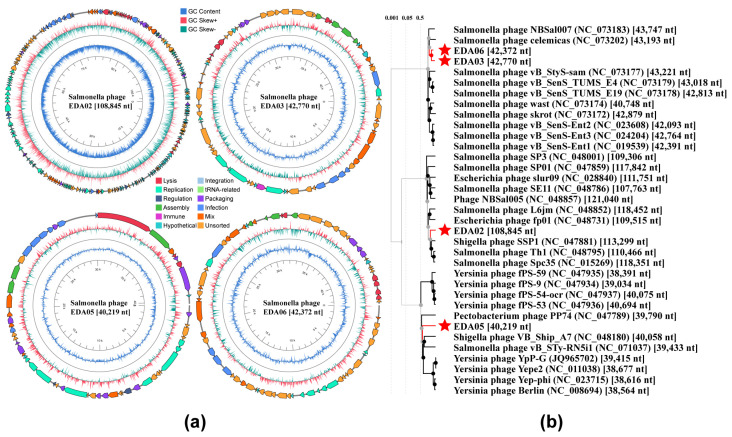
Comparative genomic organization and phylogenetic analysis of the isolated *Salmonella* bacteriophages. (**a**) Circular genome maps of bacteriophages EDA02, EDA03, EDA05, and EDA06. Open reading frames (ORFs) are shown as colored arrows and annotated according to predicted functional categories, including DNA replication, structural proteins, packaging, host lysis, regulation, and hypothetical proteins. Inner rings represent GC content and GC skew across each genome. Genome sizes are indicated at the center of each map. (**b**) Phylogenetic tree based on whole-genome sequence comparisons of EDA02, EDA03, EDA05, and EDA06 with reference bacteriophages infecting *Salmonella*, Escherichia, Shigella, Yersinia, and Pectobacterium. The tree was constructed using ViPTree and branch support values are indicated. The isolated phages characterized in this study are highlighted with a red star, showing their evolutionary relationships with closely related members of the Caudoviricetes class.

**Figure A1:**
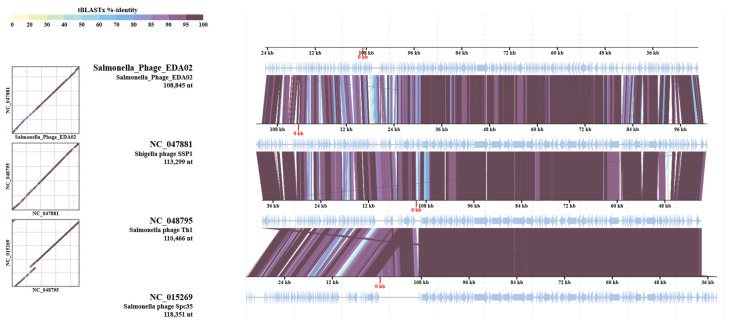
Comparative whole-genome alignment of *Salmonella* phage EDA02 with related bacteriophages. The genome of *Salmonella* phage EDA02 (108,845 bp) was aligned against reference phages including Shigella phage SSP1 (NC_047881), *Salmonella* phage Th1 (NC_048795), and *Salmonella* phage SPC35 (NC_015269) using BLAST-based genome comparison. Colored blocks indicate regions of sequence similarity, with the color gradient representing percentage identity (from low to high, as shown in the scale bar). Gene orientation and organization are displayed above each genome, highlighting a high degree of genomic synteny and conserved architecture between EDA02 and closely related phages, with localized regions of divergence.

**Figure A2:**
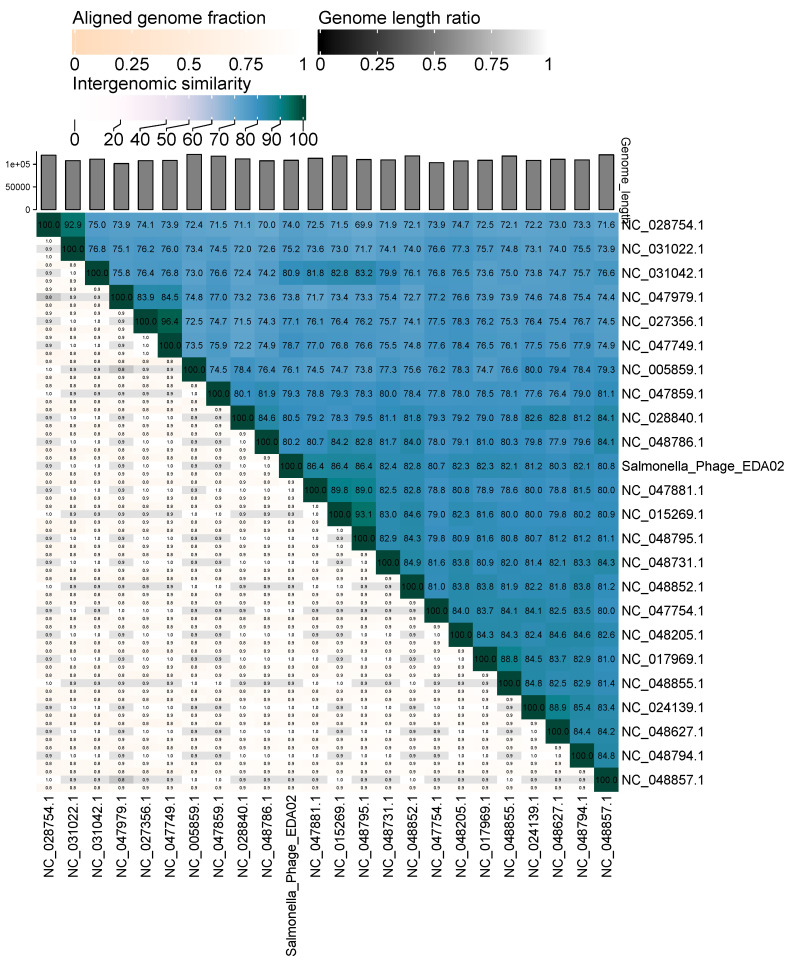
Intergenomic similarity analysis of *Salmonella* phage EDA02 and related bacteriophages. Pairwise whole-genome comparisons were performed to assess intergenomic similarity between *Salmonella* phage EDA02 and closely related phages (NC accessions shown). The heatmap displays percentage nucleotide identity, with darker colors indicating higher similarity, while values along the diagonal represent self-comparisons (100%). Bar plots above the matrix indicate the aligned genome fraction and genome length ratio for each comparison. Overall, EDA02 shows high genomic relatedness and substantial genome coverage with several reference phages, supporting its classification within a closely related *Salmonella* phage cluster while retaining distinguishable genomic divergence.

The complete genomes of the four *Salmonella* phages were assembled as single high-quality contigs and annotated using the Pharokka pipeline. All genomes exhibited the modular organization typical of Straboviridae-like caudoviricetes, comprising functional clusters involved in DNA replication and repair, nucleotide metabolism, structural assembly, and host lysis. Genomic screening using Pharokka, PhageScope, PHROGs, UniProt, and Pfam did not identify genes associated with lysogeny, virulence, or antimicrobial resistance. Resistance gene profiling of the bacterial host isolates using AMRFinderPlus and ResFinder identified multiple resistance determinants consistent with the observed phenotypic antimicrobial resistance profiles. Virion morphology was inferred from conserved structural gene modules and phylogenomic classification using ViPTree; however, transmission electron microscopy (TEM) was not performed. Whole-genome sequencing showed that *Salmonella* phage EDA02 assembled as a single circular contig representing a complete double-stranded DNA genome of 108,845 bp with a single predicted tRNA gene (Figure 7a). Approximately 180 coding sequences (CDSs) were predicted, classified into the canonical modular structure of myophages including DNA replication and repair proteins (DNA polymerase, DnaB-like helicase, NAD-dependent DNA ligase, RNA polymerase, transcriptional regulators, NrdA-like ribonucleotide reductase), structural and morphogenesis genes (capsid proteins, head maturation proteins, portal protein, baseplate and tail tube proteins, tail fiber proteins and two tail assembly chaperons). A substantial proportion of CDSs were assigned putative functions based on homology, while the remainder were annotated as hypothetical proteins, consistent with typical phage genomes. ViPTree comparative genomics positioned EDA02 within the family of Straboviridae and grouped EDA02 with *Salmonella* myophages, including vB SENS-Ent3, vB SENS-TMU5, and Th1, but in a separate branch in the clade (Figure 7b). Intergenomic comparison using the VIRIDIC pairwise comparison exhibited similarity values ranging from 71% to 85%, which is below the ICTV species demarcation threshold of 95% [39], thereby supporting the classification of phage EDA02 as a novel species within the family Straboviridae (Appendix Figure A2). Large conserved syntenic blocks of *Salmonella* phages SSP1 (NC_047881), Th1 (NC_048795), and Spc35 (NC_015269) were shown by genome-wide tBLASTx alignments between this phage and related *Salmonella* phages and are interspersed with more variable genomic islands of hypothetical proteins, also supporting its divergence (Appendix Figure A1).

**Figure A3:**
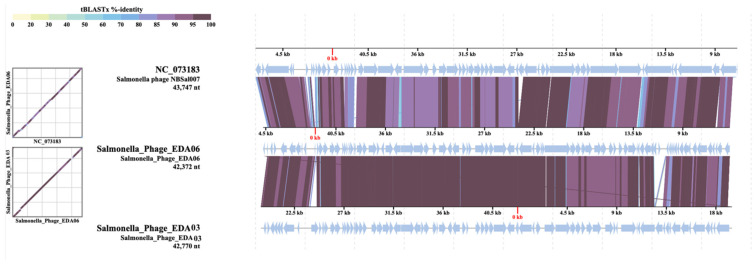
Comparative whole-genome BLAST analysis of *Salmonella* phages EDA03 and EDA06 and closely related *Salmonella* bacteriophages. The complete genomes of *Salmonella* phages EDA03 (42,770 bp) and EDA06 (42,372 bp) were aligned with the closely related *Salmonella* phage NBSal007 (NC_073183; 43,747 bp) using BLAST-based genome comparison. Colored blocks represent regions of nucleotide similarity, with the color scale indicating BLAST percentage identity. Predicted open reading frames and gene orientation are displayed above each genome. Both EDA03 and EDA06 exhibit high levels of genomic synteny and extensive sequence conservation with NBSal007 and with each other, interspersed with limited variable regions, highlighting their close evolutionary relationship while supporting their distinction as separate phage species within the same genus.

**Figure A4:**
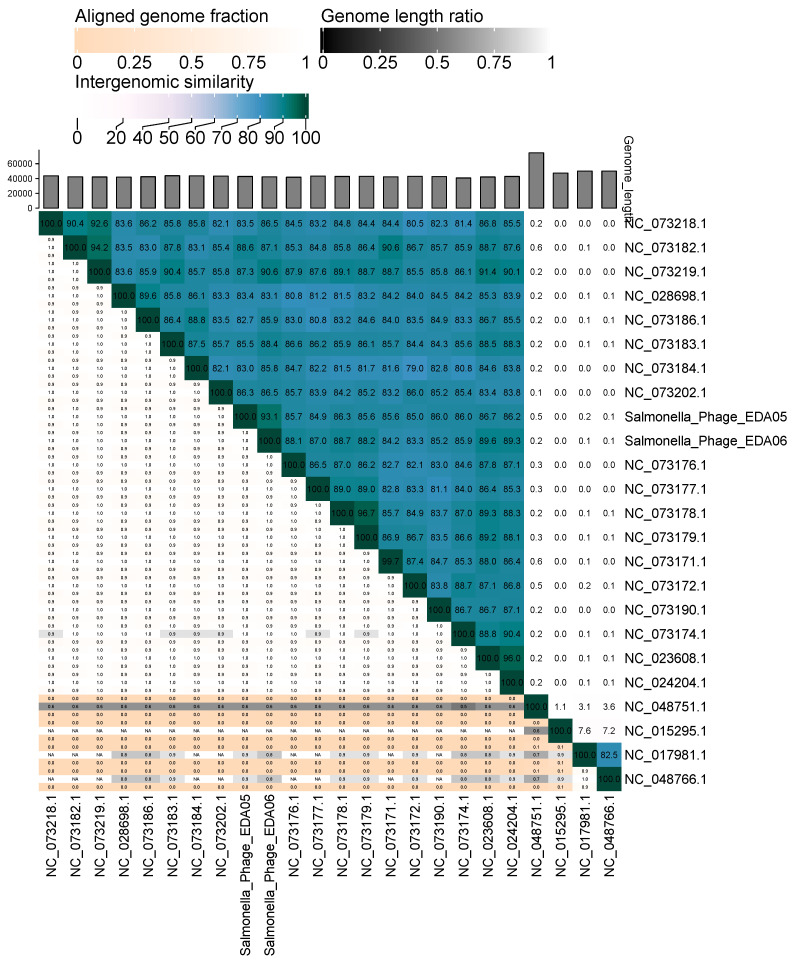
Intergenomic similarity analysis of *Salmonella* phages EDA03 and EDA06 within a cluster of related *Salmonella* bacteriophages. Pairwise whole-genome comparisons were performed between *Salmonella* phages EDA03 and EDA06 and closely related reference phages (NC accessions shown) using an intergenomic similarity approach (e.g., VIRIDIC). The heatmap depicts percentage nucleotide identity, with darker colors indicating higher similarity, while diagonal values correspond to self-comparisons (100%). Bars above the matrix represent the aligned genome fraction and genome length ratio for each comparison. EDA03 and EDA06 exhibit high intergenomic similarity with members of the same phage cluster (≈80–90%) but remain below the 95% species demarcation threshold recommended by ICTV, supporting their classification as distinct, novel bacteriophage species within an established *Salmonella* phage genus.

The genomes of phages EDA03 and EDA06 were assembled as complete dsDNA genomes of 42,770 bp and 42,372 bp, respectively, and contained approximately 55–60 predicted ORFs grouped into conserved functional units (Figure 7a). Both genomes encode usual myophage structural proteins, such as major head protein, head–tail adaptor, capsid decoration (Hoc-like) proteins, tail completion/Neck1 proteins, tail terminator, multiple minor tail proteins, tail assembly chaperones, and long tape-measure proteins, and comprise a complete virion morphogenesis module. Also identified were genes associated with adsorption, like tail fibers and tail spikes. DNA polymerase, helicase-primase enzymes, exonucleases, endonucleases, binding proteins of ssDNA, and transcriptional regulators were replication-associated ORFs. A holin, Rz/Rz-like spanin, and endolysin made up lysis modules. Additional genes associated with DNA repair and superinfection immunity were identified, including a predicted anti-repressor protein (Ant). However, the presence of an anti-repressor homolog alone was not interpreted as evidence of lysogeny, because no integrase, excisionase, site-specific recombinase, or CI-like repressor genes were detected in either genome. Proteomic clustering with ViPTree (Figure 7b) assigned both phages to the family Straboviridae and placed them in proximity to *Salmonella* phage SPN3US and Erwinia phages vB_EamM_Asesino. tBLASTx comparison revealed extensive synteny and high conservation of the majority of the genome (Appendix Figure A3). VIRIDIC analysis demonstrated that EDA03 and EDA06 shared >93% intergenomic similarity while remaining below the ICTV species demarcation threshold of 95% (Appendix Figure A4). Comparative genome analysis identified localized regions of divergence involving hypothetical proteins and putative host interaction genes, together with minor differences in genome length and gene content.

In addition, whole-genome sequencing of *Salmonella* Phage EDA05 demonstrated that it had a small circular, double-stranded DNA genome of 40,219 bp, and contained approximately 55 predicted coding sequences (Figure 7a). The genome showed a characteristic modular structure of small myophages, which consists of structural and morphogenesis proteins (major head protein, head–tail adaptor, head assembly proteins, multiple tail proteins, and a complete tail fiber assembly module), DNA replication and nucleotide metabolism proteins, including DNA polymerase I, ATP-dependent DNA ligase, exonucleases, and endonucleases, and a complete lysis module, including holin and Rz/Rz1-like spanin. Several accessory genes associated with adsorption and host interaction were also identified, including a predicted tail fiber protein and a host-range/adsorption protein.

**Figure A5:**
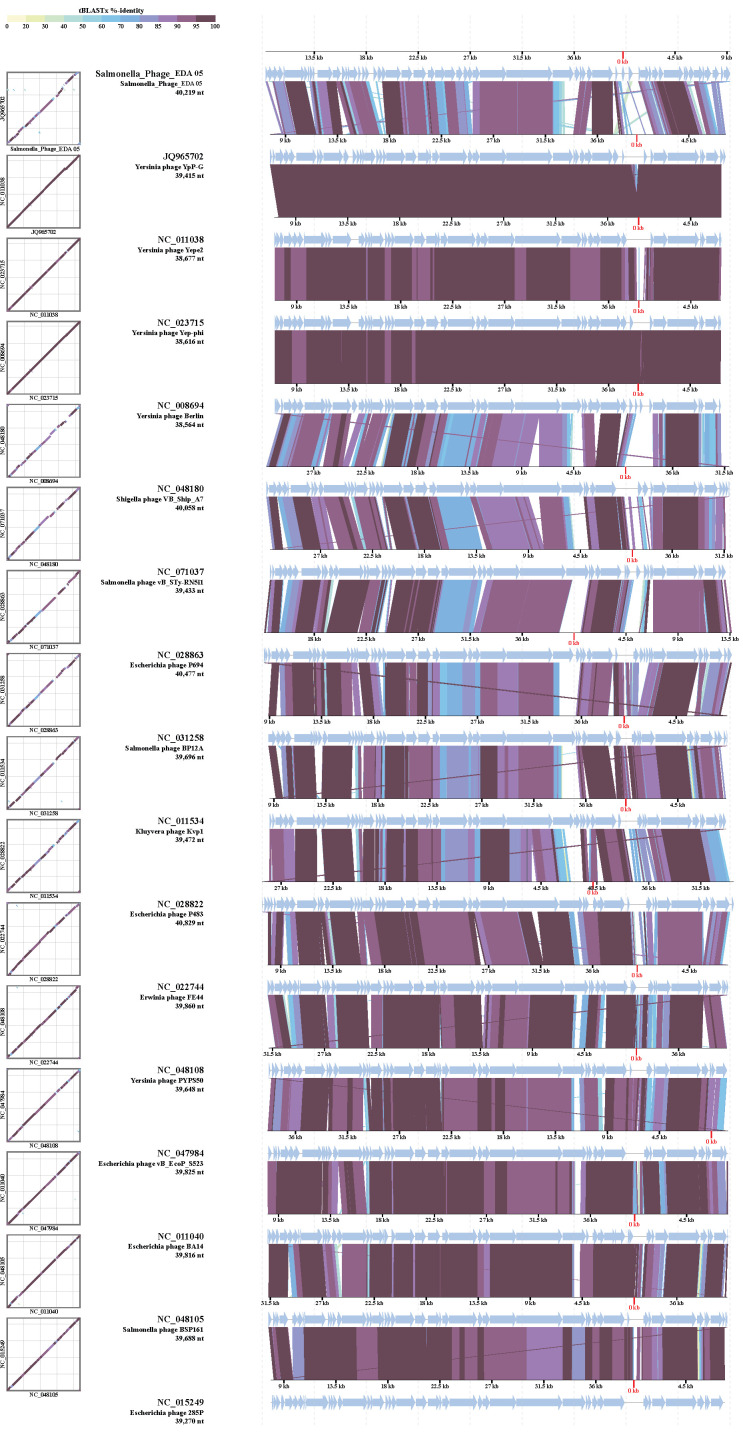
Whole-genome BLAST comparison of *Salmonella* phage EDA05 with related enterobacterial bacteriophages. The complete genome of *Salmonella* phage EDA05 (40,219 bp) was aligned against representative phages infecting *Yersinia*, *Shigella*, *Escherichia*, *Klebsiella*, *Erwinia*, and *Salmonella* using BLAST-based comparative genomics. Colored alignment blocks indicate regions of nucleotide similarity, with the color scale representing BLAST percentage identity. Gene organization and transcriptional orientation are shown above each genome. The analysis reveals extensive genome synteny with conserved structural and replication modules, interspersed with variable regions, highlighting the close evolutionary relationship of EDA05 to other enterobacterial phages while supporting its genomic distinctiveness.

**Figure A6:**
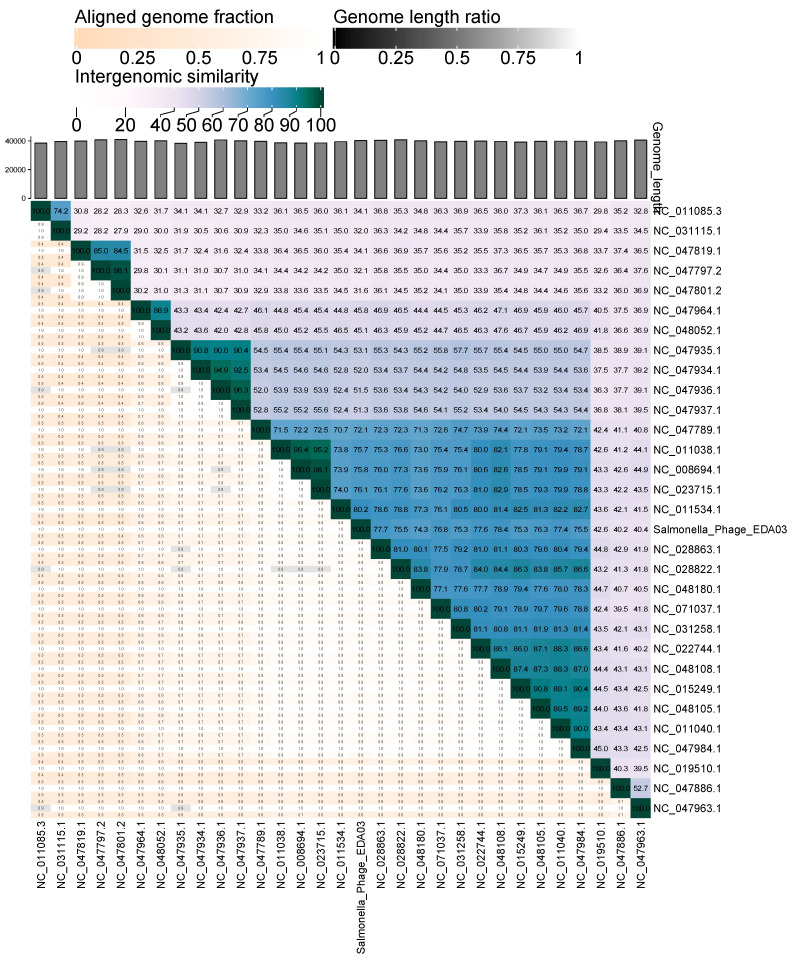
Intergenomic similarity matrix of *Salmonella* phage EDA05 and related enterobacterial bacteriophages. Pairwise whole-genome comparisons were conducted between *Salmonella* phage EDA05 and closely related bacteriophages (NC accessions shown) using intergenomic similarity analysis. The heatmap displays percentage nucleotide identity, with darker colors indicating higher similarity, while diagonal values represent self-comparisons (100%). Bars above the matrix indicate the aligned genome fraction and genome length ratio for each comparison. EDA05 shows high genomic relatedness to several reference phages but remains below the 95% species demarcation threshold recommended by ICTV, supporting its classification as a novel bacteriophage species within an established genus.

ViPTree whole-genome comparative proteomic analysis placed EDA05 within the family Straboviridae, clustering with small-genome enterobacterial myophages, including *Salmonella* phage YpP-G (JQ965702) and related phages such as YpP-G (NC_011038), YpP-G2 (NC_023715), and YpP-Ali (NC_023715), while forming a distinct side branch within the same clade (Figure 7b). This taxonomic placement was further supported by VIRIDIC analysis, which revealed intergenomic similarity values ranging from 72% to 87%, well below the 95% species demarcation threshold (Appendix Figure A6). Whole-genome comparisons additionally demonstrated conserved synteny with related enterobacterial phages (Appendix Figure A5). Notably, comparative genomic analysis indicated that EDA05 clusters with enterobacterial phages reported to infect multiple genera, including Yersinia and Shigella. Despite phylogenetic relatedness to enterobacterial phages reported to infect multiple genera, EDA05 exhibited a relatively narrow host range within the tested *Salmonella* isolate panel, potentially reflecting differences in receptor-binding proteins and adsorption-associated domains.

Together, comparative genomic analyses placed all four phages within the family Straboviridae, with intergenomic similarity values below the 95% ICTV species demarcation threshold relative to their closest reference phages. No genes associated with lysogeny, virulence, or antimicrobial resistance were detected. A comparative summary of the principal phenotypic and genomic characteristics of the four isolated phages is presented in Table 1.

### 3.7. Comparative Summary of Phage Characteristics

To facilitate comparison among the four isolated bacteriophages, their principal phenotypic and genomic characteristics are summarized in Table 1. The table integrates key parameters relevant to phage characterization and biocontrol potential, including genome size, host range, replication kinetics, physicochemical stability, genomic lifestyle, and taxonomic status. Together, these characteristics highlight both the shared properties and the distinct biological features of EDA02, EDA03, EDA05, and EDA06, providing a concise overview of their potential suitability for future phage-based applications against multidrug-resistant *Salmonella enterica* subspecies *enterica* serovar Enteritidis.

**Table 1:** Comparative summary of phenotypic and genomic characteristics of the isolated bacteriophages.

Feature	EDA02	EDA03	EDA05	EDA06
Genome size (kb)	108.8	42.8	40.2	42.4
Host range (% of tested strains lysed)	17% (3/18)	72% (13/18)	17% (3/18)	67% (12/18)
Latent period (min)	30	40	30	40
Burst size (PFU/cell)	52	6.0	150	7.0
Thermal stability range	Stable up to 50 °C	Stable up to 50 °C	Stable up to 50 °C	Stable up to 50 °C
pH stability range	4.7–11.3	4.7–10.3	4.7–10.3	3.7–11.3
Genomic lifestyle	Lytic	Lytic	Lytic	Lytic
Taxonomic status	Novel species	Novel species	Novel species	Novel species

## 4. Discussion

In this study, four novel bacteriophages, EDA02, EDA03, EDA05, and EDA06, targeting multidrug-resistant (MDR) *Salmonella* Enteritidis were successfully isolated from untreated sewage and poultry effluents in Lebanon. It is important to note that all bacterial isolates used in this study belong to ST11, reflecting the dominant outbreak-associated lineage in the region. Accordingly, the findings of this study should be interpreted within the context of ST11-specific host–phage interactions rather than representing the full diversity of *Salmonella* serovars. A comparative summary of their key phenotypic and genomic characteristics is presented in Table 1. Wastewater environments serve as prolific sources of virulent phages due to the constant exposure to bacterial hosts under anthropogenic antimicrobial pressure, driving the dynamic adaptation of the local viral microbiome [43]. An effective phage candidate for biocontrol should ideally combine species-level specificity with activity across diverse strains within the target species [44]. The lytic activity of EDA03 and EDA06 within the tested panel suggests that they may be promising candidates for inclusion in phage cocktails, providing comparatively broad intra-serovar coverage against MDR *Salmonella* within the isolate set examined [45]. Despite their close genomic relatedness, EDA03 and EDA06 displayed distinct host range and lytic characteristics. Combining phages with complementary host ranges may reduce the emergence of bacterial resistance and broaden antibacterial coverage [46,47]. In particular, extending phage selection across multiple *Salmonella* serovars will be essential for developing broadly applicable biocontrol formulations.

The interplay between AMR and phage susceptibility in *Salmonella* is a complex area of study. Our findings demonstrate that even highly resistant clinical isolates, such as those resistant to fluoroquinolones and cephalosporins, remain susceptible to these novel phages [48]. In addition, plasmid content may contribute to variability in phage susceptibility within the ST11 isolate panel. Several isolates carried IncFIB(S), IncFII(S), and IncI1-I(Alpha) plasmids, which are known to encode functions such as restriction–modification systems, superinfection exclusion mechanisms, and surface structure modifications. These plasmid-encoded features may influence phage adsorption efficiency and intracellular replication, thereby contributing to differences in lytic activity observed across isolates. Although plasmid–phage interactions were not directly tested in this study, this represents an important area for future investigation. Research has shown that the development of phage resistance can result in a high fitness cost for the bacteria, potentially increasing sensitivity to antibiotics or even restoring susceptibility to previously ineffective drugs [49,50].

Quantitative parameters derived from one-step growth curves represent critical determinants of phage therapeutic performance. However, the bacteriolytic dynamics observed in this study require careful interpretation. Although CFU and PFU quantification corroborated the OD_600_-based trends and confirmed substantial early reductions in viable bacterial populations, the re-emergence of bacterial growth at later time points indicates that suppression was not uniformly sustained. This regrowth is consistent with the emergence of phage-resistant subpopulations or incomplete lysis under reduced infection pressures, particularly at lower MOIs. Such phenomena are well documented in phage–host systems and may arise through multiple mechanisms, including receptor modification or loss, altered surface accessibility, reduced adsorption efficiency, or activation of intracellular bacterial defense pathways. In this context, phage replication dynamics further inform therapeutic potential. Previous studies have reported substantial variability in burst size among *Salmonella* phages. Reported values range from relatively low values such as phage ZCSE9 (~20 PFU per infected cell) to moderate levels exemplified by D5lw (~37 PFU per infected cell) and S4 (~41 PFU per infected cell) and extending to high-yield phages such as vB_Sal_M467 (~159 PFU per infected cell) and vB_SalM_WW2 (~150 PFU per infected cell) [23,50,51,52]. In this context, phage EDA05 exhibited a comparably high replication capacity, with a burst size of 150 PFU per infected cell, while EDA02 demonstrated intermediate productivity (52 PFU per cell). Conversely, phages EDA03 and EDA06 displayed relatively low burst sizes (6–7 PFU per cell), which likely reflect constraints in intracellular replication efficiency or host–phage interaction dynamics. Notably, phages with broader host ranges may exhibit reduced replication efficiency within individual hosts, reflecting a trade-off between host adaptability and progeny production [53].

Despite their lower burst sizes, EDA03 and EDA06 retained infectivity across multiple MDR isolates, indicating that effective bacterial suppression depends on multiple interacting factors, including adsorption efficiency, host range breadth, and replication dynamics. The integration of CFU and PFU measurements in this study strengthens the interpretation of lytic activity, while also highlighting the dynamic and non-linear nature of phage-mediated bacterial control [23]. Importantly, the observation of sustained or increasing phage titers despite partial bacterial regrowth does not necessarily represent a biological contradiction. In several phage–host systems, high extracellular PFU concentrations may coexist with renewed bacterial growth due to the emergence and expansion of resistant or phenotypically tolerant bacterial subpopulations following the initial lytic phase. In the present study, phages such as EDA05 achieved titers approaching 10^11^ PFU/mL, reflecting efficient replication within susceptible bacterial populations during the early phase of infection. However, subsequent increases in bacterial density observed after approximately 5 h suggest that a subset of surviving cells was able to proliferate despite the continued presence of abundant phage particles. Similar regrowth dynamics have been reported in *Salmonella* and other enterobacterial phage systems, where bacterial recovery occurs despite sustained phage amplification due to receptor modification, reduced adsorption efficiency, phase variation, or activation of intracellular bacterial defense mechanisms [23,47,52,54]. These findings further support the rationale for developing multi-phage cocktail formulations capable of reducing resistance emergence and improving long-term bacterial suppression.

Phage stability across a wide range of pH and temperatures is crucial for practical application. As shown in Figure 6, a marked decline in infectivity was observed at 70 °C, although residual activity was retained by some phages, consistent with reports that elevated temperatures can denature capsid structural proteins and damage viral nucleic acids [55]. Additionally, a sharp decline in activity was observed at pH 12, likely due to the dissociation of capsid proteins caused by high concentrations of hydroxyl ions in the solution [56]. However, the stability of these phages between pH 4.7 and 10.3, with minor phage-specific extensions beyond this range, and up to 50 °C ensures their functionality in diverse environments, from the human gastrointestinal tract to industrial food-processing matrices. This physicochemical resilience also supports their potential integration into food-processing pipelines, including surface decontamination, equipment sanitation, and cold-chain-associated biocontrol applications.

The genomic analysis of phages EDA02, EDA03, EDA05, and EDA06 revealed significant insights into their genetic composition and safety. Comparative genomic and phylogenetic analyses (Figure 7) placed the isolates within the Straboviridae family, closely related to other virulent *Salmonella* phages [57], although structural classification remains to be confirmed by direct electron microscopy. Despite phylogenetic relatedness to enterobacterial phages infecting multiple genera, EDA05 exhibited a relatively narrow host range within the tested *Salmonella* panel. Importantly, the total absence of genes related to lysogeny (integrases, repressors), virulence factors, or AMR genes supports their lytic nature based on genomic features. To increase the robustness of this conclusion, genomic safety screening was cross-validated across multiple annotation and conserved-domain platforms, including Pharokka, PhageScope, PHROGs, UniProt, and Pfam. Although anti-repressor homologs were annotated in EDA03 and EDA06, these proteins were interpreted cautiously and not considered sufficient evidence of a temperate lifestyle in the absence of integrases, excisionases, recombinases, or CI-like repressors. Accordingly, their presence was considered an isolated annotation rather than a marker of lysogeny. The absence of canonical lysogeny-associated genes and known antimicrobial resistance determinants supports the lytic profile of these phages and is consistent with desirable preliminary characteristics for future biocontrol development [58]. However, regulatory suitability for applied use would require additional evaluation, including efficacy, safety, formulation stability, manufacturing quality control, and jurisdiction-specific regulatory review.

Compared to previously reported *Salmonella* bacteriophages, the isolates characterized in this study present several distinguishing features. First, the combination of complementary host range profiles (EDA03/EDA06) with high replication efficiency (EDA05) supports rational cocktail design strategies. Second, the isolates demonstrate robust physicochemical stability across a broad range of pH and temperature conditions, enhancing their applicability in food-processing environments. Finally, their taxonomic novelty (<95% intergenomic similarity) highlights the presence of previously uncharacterized phage diversity in Middle Eastern wastewater systems. Collectively, these features distinguish the present isolates from previously reported *Salmonella* phages and reinforce their potential for translational biocontrol applications.

The taxonomic placement of these isolates within the Straboviridae family, coupled with their distinct intergenomic distances (<95% similarity to RefSeq relatives), underscores the untapped viral diversity present in Middle Eastern wastewater. These findings provide a genomically verified foundation for future phage-based biocontrol strategies against MDR *Salmonella* in the region [59].

The increasing prevalence of MDR *Salmonella enterica* highlights the urgent need for alternative antimicrobial strategies capable of reducing dependence on conventional antibiotics. In this context, lytic bacteriophages represent promising targeted biocontrol agents because they can selectively eliminate pathogenic bacteria while minimizing disruption of beneficial microbiota. The genomic safety profile of the isolated phages further supports their potential suitability for therapeutic and food-safety applications. Future work should focus on phage cocktail optimization, evaluation in food matrices and animal infection models, and assessment of synergistic interactions between phages and antibiotics as part of integrated antimicrobial stewardship strategies.

This study has several limitations. First, the host panel consisted exclusively of *S.* Enteritidis ST11 isolates derived from a single surveillance program and originating from human clinical samples. While this reflects a clinically relevant and epidemiologically dominant lineage, it introduces a degree of genetic and ecological homogeneity and raises the possibility of clonal relatedness among isolates, thereby limiting the generalizability of the findings to other *Salmonella* serovars, sequence types, or environmental reservoirs such as poultry or wastewater-associated strains. Future studies should therefore expand the host panel to include multiple serovars (e.g., *S.* Typhimurium), additional sequence types, and isolates from diverse ecological sources in order to better evaluate the breadth and translational applicability of the identified phages. In addition, the host range panel was limited to 18 regional MDR isolates. An additional limitation of this study is that the *Salmonella enterica* serovar Enteritidis isolates used here did not exhibit significant biofilm-forming capacity under the tested in vitro conditions. Accordingly, the bacteriolytic activity reported herein primarily reflects phage efficacy against planktonic bacterial populations rather than established biofilms. Because biofilm formation is highly dependent on environmental conditions, surface properties, and strain-specific characteristics, the absence of biofilm formation under the tested conditions should not be generalized to all *Salmonella* Enteritidis isolates.

Second, transmission electron microscopy (TEM) was not performed; therefore, virion morphology was inferred from plaque phenotype, comparative genomics, and phylogenomic classification rather than direct ultrastructural observation. Although phylogenomic classification using ViPTree together with conserved structural gene architecture provides strong taxonomic inference consistent with Caudoviricetes morphology, direct visualization remains necessary for definitive morphological classification and assessment of phage preparation purity. Future studies will incorporate TEM-based imaging to validate virion structure and confirm the absence of contaminating virus-like particles. In addition, genome assembly was performed using Oxford Nanopore long-read sequencing without short-read hybrid polishing. Although the assemblies were obtained as high-quality single contigs with sufficient depth, residual base-calling errors, particularly in homopolymeric regions, cannot be fully excluded. Future studies integrating hybrid sequencing approaches would further improve nucleotide-level accuracy and annotation confidence. Third, although bacteriolytic activity was validated using CFU and PFU quantification, the emergence of bacterial regrowth at later time points indicates the likely development of phage-resistant subpopulations or incomplete lysis under some conditions, and the underlying mechanisms were not investigated in the present study. Future work should characterize the mechanisms underlying bacterial regrowth and phage resistance. A major limitation of this study is that the biocontrol efficacy of the phages was evaluated exclusively under in vitro conditions. While these assays provide controlled and reproducible insights into phage–host dynamics, they do not fully capture the complexity of biologically relevant environments such as food matrices or in vivo systems. Accordingly, the present findings should be interpreted as foundational, preclinical evidence of phage efficacy rather than direct translational validation. Future studies will be required to evaluate phage performance in complex systems, including poultry-associated food matrices and in vivo infection models, in order to assess their stability, efficacy, and safety under realistic application conditions. In addition, the efficacy of these bacteriophages against biofilm-forming *Salmonella* under optimized or stress-induced conditions should be further investigated. No canonical depolymerase-encoding genes were identified in the analyzed genomes. As depolymerases are often associated with biofilm matrix degradation, their absence may limit direct antibiofilm activity and suggests that efficacy against structured communities may rely on indirect mechanisms. Future studies should evaluate biofilm activity and explore combination strategies to enhance performance.

Although the present study was conducted under controlled in vitro conditions, it provides a foundational framework for future translational investigations in food matrices and in vivo systems. Collectively, these findings support the development of scalable, antibiotic-free interventions against MDR foodborne pathogens, particularly in understudied regions such as the Middle East.

## 5. Raw Data Availability

Raw sequencing data for *Salmonella* phages EDA02, EDA03, EDA05 and EDA06 and the associated bacterial isolates have been deposited in publicly accessible databases. The genomic sequences of *Salmonella* phages EDA02, EDA03, EDA05 and EDA06 are available in the NCBI GenBank, under the accession numbers, respectively, PX630757, PX630758, PX630759 and PX630760.

## Data Availability

The original contributions presented in this study are included in the article. further inquiries can be directed to the corresponding authors.

## Appendix A

**Table A1:** Antimicrobial susceptibility profiles and resistance classification of multidrug-resistant *Salmonella enterica* serovar Enteritidis ST11 isolates used in this study.

Species	ST	Source (Site)	Plasmids	Ceftriaxone CRO (R ≥ 4) VITEK	Meropenem (R ≥ 4)	Ampicillin (R ≥ 32)	Amoxicillin-Clavulanic Acid—Amoclan (≥8) EUCAST	Gentamicin (R > 2) EUCAST	Ciprofloxacin (R ≥ 1)	Nalidixic Acid (≥32) VITEK	Tetracycline (R ≥ 16) VITEK	Chloramphenicol (R ≥ 32) BMD	Colistin (>2) BMD	Trimethoprim-Sulfamethoxazole (≥4/76) VITEK	Azithromycin (≥32) BMD	Profile
220	11	Human stool	IncFIB(S)	>64 R	>16 R	<0.25 S	<2 S	>16 R	4 R	64 R	>16 R	64 R	1 S	>512 R	>512 R	XDR
222	11	Human stool	IncFIB(S)	<1 S	<0.25 S	<0.25 S	<2 S	>16 R	4 R	>512 R	20 S	8 S	8 R	512 R	64 R	MDR
227	11	Human stool	IncFIB(S)	<1 S	<0.25 S	<0.25 S	<2 S	<1 S	4 R	>512 R	>16 R	16 S	1 S	>512 R	16 S	MDR
228	11	Human stool	IncFIB(S), IncFII(S)	<1 S	<0.25 S	6 R	32 R	>16 R	4 R	>512 R	>16 R	64 R	1 S	>512 R	64 R	MDR
236	11	Human stool	IncFIB(S), IncFII(S)	<1 S	<0.25 S	<2 S	<2 S	>16 R	<0.25 S	>512 R	<1 S	64 R	0.5 S	>512 R	32 R	MDR
240	11	Human stool	IncFIB(S), IncFII(S)	<1 S	<0.25 S	<2 S	<8 S	<1 S	<0.25 S	>32 R	<1 S	64 R	<0.25 S	>512 R	2 S	MDR
241	11	Human stool	IncFIB(S), IncFII(S)	<1 S	<0.25 S	<2 S	<8 S	>16 R	<0.25 S	>32 R	<1 S	32 R	<0.25 S	>512 R	4 S	MDR
242	11	Human stool	IncFIB(S), IncFII(S)	<1 S	<0.25 S	<2 S	<8 S	>16 R	<0.25 S	>32 R	<1 S	16 S	16 R	<20 S	2 S	MDR
256	11	Fish	IncFIB(S)	1 S	0.25 S	2 S	8 R	>16 R	0.25 S	32 R	16 R	128 R	1 S	16 R	4 S	MDR
262	11	Stool	IncFIB(S),IncFII(S)	1 S	0.25 S	2 S	8 R	>16 R	>16 R	32 R	16 R	32 R	0.5 S	16 R	4 S	MDR
271	11	Stool	IncFIB(S),IncFII(S)	1 S	0.25 S	2 S	8 R	>16 R	>16 R	32 R	1 S	256 R	2 S	16 R	4 S	MDR
281	11	Stool	IncFIB(S),IncFII(S)	1 S	0.25 S	2 S	8 R	>16 R	>16 R	32 R	16 R	>512 R	<0.25 S	20 S	64 R	MDR
282	11	Stool	IncFIB(S), IncFII(S), IncI1-I(Alpha)	1 S	0.25 S	2 S	8 R	>16 R	>16 R	32 R	16 R	>512 R	<0.25 S	20 S	128 R	MDR
286	11	Stool	IncFIB(S), IncFII(S)	1 S	0.25 S	2 S	8 R	>16 R	>16 R	32 R	16 R	>512 R	<0.25 S	20 S	64 R	MDR
287	11	Stool	IncFIB(S), IncFII(S)	1 S	0.25 S	2 S	8 R	>16 R	>16 R	32 R	16 R	>512 R	<0.25 S	20 S	128 R	MDR
293	11	Stool	IncFIB(S), IncFII(S)	2 S	<0.25 S	6 R	8 R	>16 R	>16 R	>32 R	>16 R	16 S	<0.25 S	>512 R	2 S	MDR
294	11	Stool	IncFIB(S), IncFII(S)	3 S	<0.25 S	6 R	<2 S	>16 R	>16 R	>512 R	16 R	8 S	<0.25 S	16 R	2 S	MDR
295	11	Stool	IncFIB(S), IncFII(S), IncI1-I(Alpha)	>64 R	0.25 S	<2 S	8 R	>16 R	>16 R	>32 R	16 R	16 S	<0.25 S	16 R	4 S	MDR

Note: Values shown in red indicate resistance (R), whereas values shown in black indicate susceptibility (S) according to the corresponding breakpoint criteria. MDR = multidrug-resistant; XDR = extensively drug-resistant.

**Table A2:** EOP of *Salmonella* phages against MDR clinical isolates, calculated from triplicate plaque assays relative to the original host strain.

Strain ID	EDA02 EOP	EDA03 EOP	EDA05 EOP	EDA06 EOP
SAL281	–	0.3 ± 0.03	–	0.35 ± 0.02
SAL282	**1 ± 0.04**	–	–	–
SAL295	0.42 ± 0.03	0.51 ± 0.04	0.31 ± 0.02	0.43 ± 0.04
SAL256	–	–	**1 ± 0.04**	–
SAL227	–	0.31 ± 0.02	0.19 ± 0.02	0.31 ± 0.02
SAL220	0.35 ± 0.03	0.46 ± 0.02	–	0.28 ± 0.03
SAL222	0.32 ± 0.03	0.41 ± 0.03	–	0.32 ± 0.03
SAL262	0.37 ± 0.02	0.44 ± 0.03	–	0.34 ± 0.04
SAL228	0.31 ± 0.03	0.43 ± 0.03	–	–
SAL241	0.34 ± 0.02	0.49 ± 0.04	–	0.37 ± 0.04
SAL242	0.40 ± 0.03	0.48 ± 0.02	–	**1 ± 0.04**
SAL271	–	0.28 ± 0.02	–	0.29 ± 0.03
SAL240	–	**1 ± 0.05**	–	0.41 ± 0.02
SAL293	0.29 ± 0.02	0.38 ± 0.03	–	0.31 ± 0.04
SAL236	–	0.43 ± 0.03	–	0.35 ± 0.02
SAL294	–	–	–	–
SAL287	–	0.44 ± 0.04	–	0.37 ± 0.03
SAL286	0.34 ± 0.04	0.43 ± 0.03	–	0.35 ± 0.04

Note: Values in bold red denote the original host strain used for isolation of the corresponding bacteriophage and serve as the reference for EOP calculation. Data are presented as mean ± SD from three independent experiments. The symbol (–) indicates absence of detectable lytic activity.

**Table A3:** Quantitative assessment of bacterial viability (CFU/mL) and phage replication (PFU/mL) during bacteriolytic assays at varying MOIs over time.

**EDA02**						**EDA05**					
**CFU**						**CFU**					
**Time (h)**	**0**	**1**	**4**	**8**	**12**	**Time (h)**	**0**	**1**	**4**	**8**	**12**
NC	3.4 × 10^7^	3.4 × 10^7^	3.5 × 10^7^	4.0 × 10^7^	4.8 × 10^7^	NC	3.4 × 10^7^	3.4 × 10^7^	3.5 × 10^7^	4.0 × 10^7^	4.8 × 10^7^
PC (282)	4.4 × 10^7^	7.1 × 10^7^	4.4 × 10^8^	7.5 × 10^8^	1.0 × 10^9^	PC (256)	1.3 × 10^8^	2.7 × 10^8^	6.1 × 10^8^	9.8 × 10^8^	1.2 × 10^9^
MOI 0.01	4.4 × 10^7^	7.2 × 10^7^	1.8 × 10^8^	1.0 × 10^8^	3.2 × 10^8^	MOI 0.01	8.1 × 10^7^	8.3 × 10^7^	8.7 × 10^7^	1.0 × 10^8^	1.4 × 10^8^
MOI 0.1	4.4 × 10^7^	6.9 × 10^7^	1.1 × 10^8^	1.2 × 10^8^	4.2 × 10^8^	MOI 0.1	6.5 × 10^7^	8.3 × 10^7^	6.9 × 10^7^	9.3 × 10^7^	1.8 × 10^8^
MOI 1	4.6 × 10^7^	6.1 × 10^7^	6.3 × 10^7^	1.6 × 10^8^	4.3 × 10^8^	MOI 1	6.5 × 10^7^	9.3 × 10^7^	6.8 × 10^7^	1.0 × 10^8^	3.1 × 10^8^
MOI 10	4.3 × 10^7^	4.6 × 10^7^	4.7 × 10^7^	1.4 × 10^8^	4.4 × 10^8^	MOI 10	6.0 × 10^7^	8.5 × 10^7^	7.1 × 10^7^	1.6 × 10^8^	2.1 × 10^8^
**PFU**						**PFU**					
**Time (h)**	**0**	**1**	**4**	**8**	**12**	**Time (h)**	**0**	**1**	**4**	**8**	**12**
MOI 0.01	1.0 × 10^6^	5.6 × 10^7^	1.2 × 10^11^	1.3 × 10^11^	1.4 × 10^11^	MOI 0.01	1.2 × 10^6^	1.1 × 10^8^	3.0 × 10^10^	2.3 × 10^11^	2.2 × 10^11^
MOI 0.1	1.1 × 10^7^	5.4 × 10^8^	2.8 × 10^10^	2.8 × 10^10^	2.8 × 10^10^	MOI 0.1	1.2 × 10^7^	1.2 × 10^9^	1.7 × 10^10^	1.6 × 10^11^	1.7 × 10^11^
MOI 1	1.2 × 10^8^	4.9 × 10^9^	5.0 × 10^9^	5.1 × 10^9^	5.0 × 10^9^	MOI 1	1.2 × 10^8^	1.1 × 10^10^	1.7 × 10^10^	1.7 × 10^10^	1.8 × 10^10^
MOI 10	1.1 × 10^10^	4.4 × 10^11^	6.0 × 10^11^	5.1 × 10^11^	5.0 × 10^11^	MOI 10	1.2 × 10^10^	7.0 × 10^10^	1.3 × 10^11^	1.4 × 10^11^	1.3 × 10^11^
**EDA03**						**EDA06**					
**CFU**						**CFU**					
**Time (h)**	**0**	**1**	**4**	**8**	**12**	**Time (h)**	**0**	**1**	**4**	**8**	**12**
NC	3.4 × 10^7^	3.4 × 10^7^	3.5 × 10^7^	4.0 × 10^7^	4.8 × 10^7^	NC	3.4 × 10^7^	3.4 × 10^7^	3.5 × 10^7^	4.0 × 10^7^	4.8 × 10^7^
PC (240)	1.3 × 10^8^	2.4 × 10^8^	5.4 × 10^8^	9.3 × 10^8^	9.9 × 10^8^	PC (242)	1.3 × 10^8^	2.5 × 10^8^	5.2 × 10^8^	9.8 × 10^8^	1.2 × 10^9^
MOI 0.01	1.3 × 10^8^	1.8 × 10^8^	2.4 × 10^8^	5.9 × 10^8^	9.0 × 10^8^	MOI 0.01	1.3 × 10^8^	1.4 × 10^8^	2.1 × 10^8^	4.0 × 10^8^	7.3 × 10^8^
MOI 0.1	1.2 × 10^8^	1.3 × 10^8^	2.2 × 10^8^	5.4 × 10^8^	9.1 × 10^8^	MOI 0.1	1.1 × 10^8^	9.0 × 10^7^	2.3 × 10^8^	3.5 × 10^8^	7.4 × 10^8^
MOI 1	1.0 × 10^8^	9.4 × 10^7^	2.1 × 10^8^	4.9 × 10^8^	9.1 × 10^8^	MOI 1	9.8 × 10^7^	7.0 × 10^7^	2.4 × 10^8^	4.3 × 10^8^	7.3 × 10^8^
MOI 10	7.9 × 10^7^	7.9 × 10^7^	1.9 × 10^8^	4.7 × 10^8^	8.6 × 10^8^	MOI 10	6.8 × 10^7^	6.2 × 10^7^	2.0 × 10^8^	3.2 × 10^8^	6.7 × 10^8^
**PFU**						**PFU**					
**Time (h)**	**0**	**1**	**4**	**8**	**12**	**Time (h)**	**0**	**1**	**4**	**8**	**12**
MOI 0.01	2.0 × 10^6^	1.6 × 10^7^	3.5 × 10^8^	4.3 × 10^8^	4.3 × 10^8^	MOI 0.01	1.5 × 10^6^	1.2 × 10^7^	4.1 × 10^8^	5.2 × 10^8^	5.3 × 10^8^
MOI 0.1	2.2 × 10^7^	1.7 × 10^8^	2.6 × 10^8^	2.6 × 10^8^	2.7 × 10^8^	MOI 0.1	1.4 × 10^7^	1.1 × 10^8^	7.3 × 10^8^	7.4 × 10^8^	7.4 × 10^8^
MOI 1	2.5 × 10^8^	1.5 × 10^9^	1.5 × 10^9^	1.5 × 10^9^	1.5 × 10^9^	MOI 1	1.4 × 10^8^	1.1 × 10^9^	1.1 × 10^9^	1.2 × 10^9^	1.1 × 10^9^
MOI 10	2.6 × 10^10^	1.4 × 10^11^	1.6 × 10^11^	1.6 × 10^11^	1.6 × 10^11^	MOI 10	1.5 × 10^10^	1.0 × 10^11^	1.1 × 10^11^	1.1 × 10^11^	1.1 × 10^11^
